# Supplementary material for: Renal Glucose Release after Unilateral Renal Denervation during a Hypoglycemic Clamp in Pigs with an Altered Hypothalamic Pituitary Adrenal Axis after Late-Gestational Dexamethasone Injection
Source: Int J Mol Sci. 2023 Aug 13;24(16):12738. doi: 10.3390/ijms241612738 (PMC10454812; doi:10.3390/ijms241612738)
Supplement: Supplementary file 1 [file ijms-24-12738-s001.zip › ijms-2494965-supplementary.pdf]

Table S1. Original data of SGN after unilateral ablation of renal nerves.

Number of animal	1	2	3	4	5	6	7	8	9	10
Body site of ablation	right	right	left	left	right	right	left	right	left	left
SGN of ablated kidney at duration of hypoglycaemia										
45′	0.0032	0.0012	0.0010	0.0002	0.0095	0.0097	0.5727	0.0894	0.4902	0.0032
60′	0.0115	0.0058	0.0055	0.0003	0.0013	0.0682	0.1540	0.0494	0.0279	0.0115
75′	0.0174	0.0056	0.0035	0.0019	0.1241	0.9722	1.0431	0.0224	0.1464	0.0174
90′	0.0062	0.0002	0.0055	0.0002	0.0754	0.1652	0.2110	0.0196	0.1726	0.0062
105′	0.0156	0.0005	0.0109	0.0031	0.0075	0.0236	0.0745	0.0505	0.0745	0.0156
SGN of non-ablated kidney at duration of hypoglycaemia										
45′	0.0190	0.0208	0.0149	0.0004	0.1001	0.0116	1.3297	0.1096	0.2482	0.0190
60′	0.0130	0.0062	0.0872	0.0011	0.0641	0.0968	0.1670	0.1613	0.5763	0.0130
75′	0.0231	0.0294	0.0154	0.0063	0.1590	0.9779	0.2437	0.0224	0.0314	0.0231
90′	0.0092	0.0027	0.0693	0.0012	0.2158	0.1629	0.2877	0.0126	0.1962	0.0092
105′	0.0044	0.0140	0.0535	0.0091	0.3181	0.0437	0.1734	0.0316	0.1948	0.0044
